# Integrated analysis of droxidopa trials for neurogenic orthostatic hypotension

**DOI:** 10.1186/s12883-017-0867-5

**Published:** 2017-05-12

**Authors:** Italo Biaggioni, L. Arthur Hewitt, Gerald J. Rowse, Horacio Kaufmann

**Affiliations:** 1 Department of Medicine, Division of Clinical Pharmacology, 560A RRB, Vanderbilt University School of Medicine, Nashville, TN 37232 USA; 2 Lundbeck LLC, 6 Parkway North, Deerfield, IL 60015 USA; 3 Department of Neurology, Dysautonomia Center, New York University School of Medicine, 530 First Avenue, Suite 9Q, New York, NY 10016 USA

**Keywords:** Autonomic nervous system, Norepinephrine, Parkinson disease

## Abstract

**Background:**

Droxidopa, a prodrug of norepinephrine, was approved for treatment of neurogenic orthostatic hypotension (nOH) due to primary autonomic disorders based on 3 randomized double-blind studies. We performed safety and efficacy analyses of this pooled dataset (*n* = 460).

**Methods:**

Efficacy was assessed using Orthostatic Hypotension Questionnaire (OHQ) scores (composite and individual items). Safety and tolerability were also examined.

**Results:**

Droxidopa improved virtually all nOH symptom scores compared with placebo, significantly reducing OHQ composite score (−2.68 ± 2.20 vs −1.82 ± 2.34 units; *P* < 0.001), dizziness/lightheadedness score (−3.0 ± 2.9 vs −1.8 ± 3.1 units; *P* < 0.001), and 3 of 5 other symptom assessments (visual disturbances, weakness, and fatigue [*P* ≤ 0.010]). Droxidopa significantly improved 3 of 4 measures of activities of daily living (standing a long time, walking a short time, and walking a long time [*P* ≤ 0.003]) and significantly increased upright systolic blood pressure (11.5 ± 20.5 vs 4.8 ± 21.0 mmHg for placebo; *P* < 0.001). Droxidopa was effective in patients using inhibitors of dopa decarboxylase (DDCI; the enzyme that converts droxidopa to norepinephrine), but its efficacy was numerically greater in non-DDCI users. Droxidopa was well-tolerated. Rates of most adverse events were similar between groups. Supine hypertension rates were low, but slightly higher in patients receiving droxidopa (≤7.9% vs ≤4.6% for placebo); patients with severe hypertension at screening were excluded from these studies.

**Conclusions:**

Droxidopa is effective for the treatment of nOH in patients with primary autonomic disorders and is generally well-tolerated. A longer trial is underway to confirm efficacy beyond the ≤2 to 10 - week period assessed in the current trials.

**Trial registration:**

ClinicalTrials.gov identifiers: NCT00782340, first received October 29, 2008; NCT00633880, first received March 5, 2008; and NCT01176240, first received July 30, 2010.

## Background

Orthostatic hypotension (OH) is a relatively common problem in the elderly, a significant cause of disability, and an independent risk factor for falls and mortality [1–5]. Neurogenic OH (nOH) is a less common but more severe form of the condition, and is the result of an impaired sympathetic nervous system response during transition to an upright posture associated with reduced norepinephrine release [6].

Despite the clinical importance of nOH, there has been a paucity of treatment options for this orphan condition [7]. For nearly 2 decades, no new treatments were introduced; in 2014, droxidopa was approved by the US Food and Drug Administration (FDA) for the treatment of symptomatic nOH caused by primary autonomic failure (Parkinson disease [PD], multiple system atrophy [MSA], pure autonomic failure), dopamine β-hydroxylase deficiency (DBHD), and nondiabetic autonomic neuropathy (NDAN) [8]. Droxidopa is a prodrug that is converted into norepinephrine by dopa decarboxylase, the same enzyme that converts levodopa into dopamine [9].

Accelerated approval by the FDA under Subpart H was based on 3 randomized controlled double-blind clinical studies that showed the efficacy of droxidopa for relief of nOH symptoms [10–13]. In Studies NOH301 and NOH306, patients were randomized to placebo or droxidopa to determine if their symptoms would improve during active treatment [11, 12]. In Study NOH302, patients treated with open-label droxidopa were randomized to continue receiving active treatment or to switch to placebo to determine if withdrawal from active treatment was associated with worsening of symptoms [10].

Cumulatively, these trials represent the largest experience to date in the treatment of nOH. Here, we analyzed the integrated data from these 3 trials. The increased number of observations provided us with a unique opportunity to perform a pooled efficacy analysis and a post hoc targeted subgroup analysis to improve our understanding of nOH and to provide physicians with more extensive information about the use of droxidopa for the treatment of patients with nOH.

## Methods

For this integrated analysis, data were included from patients who were randomized to double-blind treatment with droxidopa or placebo in 3 clinical trials (ie, each individual full analysis set) [10–13]. Patient selection and study designs for the individual studies have been published previously, and are described briefly in the following sections.

### Study patients

All three clinical trials included patients with a diagnosis of a primary neurodegenerative disease and symptomatic nOH, and shared similar inclusion and exclusion criteria. Adults ≥18 years old with nOH diagnosed using a documented decrease of ≥20 mmHg in systolic blood pressure (SBP) or ≥10 mmHg in diastolic blood pressure (DBP) within 3 min of standing were eligible for enrollment. Patients in Studies NOH301 and NOH302 were required to have a clinical diagnosis of symptomatic nOH caused by primary autonomic failure (associated with PD, MSA, or pure autonomic failure), NDAN, or DBHD [10, 11]. In Study NOH306, only patients with PD and values ≥3 for the Orthostatic Hypotension Questionnaire (OHQ; described in the following section) composite score and the Clinical Global Impression–Severity (CGI-S) rating were enrolled [12].

Exclusion criteria included the use of vasoconstrictive agents (e.g., ephedrine, dihydroergotamine, midodrine) within 2 days before study entry, and antihypertensive agents other than short-acting antihypertensives given at bedtime. Norepinephrine reuptake inhibitors (NRIs; e.g., tricyclic antidepressants) were excluded in Studies NOH301 and NOH302, but were allowed in Study NOH306. Patients with PD were allowed to continue stable doses of antiparkinsonian medications. Patients with severe hypertension (≥180/110 mmHg while seated or supine), significant systemic, hepatic, cardiac, or renal illness, or diabetes (mellitus or insipidus in Studies NOH301 and NOH 302, diabetic neuropathy in Study NOH306) were excluded from participation in these studies [10–13].

### Study designs

Study NOH301 included patients with symptomatic nOH who responded to open-label droxidopa treatment before a 1-week washout and randomization to double-blind treatment for 1 week with droxidopa or placebo [11]. In Study NOH302, patients with symptomatic nOH who responded during the open-label dose optimization period continued open-label droxidopa for 1 week before double-blind randomization to continue treatment with droxidopa or withdrawal to placebo for 2 weeks [10]. In Study NOH306, patients with symptomatic nOH associated with PD underwent double-blind titration of droxidopa or placebo over 2 weeks before receiving a maintenance dose for 8 to 10 weeks [12, 13]. For the current analyses, the differences in OHQ scores (composite and component) from baseline to 1 week of droxidopa treatment were evaluated.

### Symptomatic efficacy assessments

**Fig. 1:**
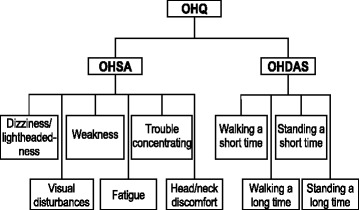
OHQ items evaluate orthostatic symptoms (OHSA; *n* = 6) and activities of daily living (OHDAS; *n* = 4) [14]. OHDAS = Orthostatic Hypotension Daily Activity Scale; OHQ = Orthostatic Hypotension Questionnaire; OHSA = Orthostatic Hypotension Symptom Assessment

In all studies, patients rated nOH symptoms and symptom impact using the OHQ (Fig. 1), which includes the 6-item Orthostatic Hypotension Symptom Assessment (OHSA) scale and the 4-item Orthostatic Hypotension Daily Activity Scale (OHDAS) [14]. The OHSA measures intensity of dizziness/lightheadedness, visual disturbance, weakness, fatigue, trouble concentrating, and head/neck discomfort (Items 1–6); the OHDAS assesses the impact of nOH symptoms on daily activities requiring standing or walking for short or long periods (Items 1–4). Each item is scored from 0 (none/no interference) to 10 (worst possible/complete interference), describing the preceding week. Composite OHSA and composite OHDAS scores averaged the scores for items rated >0 at baseline. The composite OHQ score is the average of the OHSA and OHDAS composite scores [10–13].

In addition, clinicians and patients rated the severity of nOH using a CGI-S instrument, adapted from Guy [15], on a scale from 1 (no symptoms) to 7 (severe symptoms). Based on the CGI-S rating, patients were grouped into 3 predefined symptom categories: Normal-Borderline OH (CGI-S score range 1–2), Mild-Moderate OH (CGI-S score range 3–4), and Marked OH-Most Ill with OH (CGI-S score range 5–7).

### Hemodynamic efficacy assessment

Patients underwent an orthostatic standing test at all scheduled clinic visits. Each test included measurements of blood pressure (BP) and heart rate at the end of a 10-min supine period (with the patient’s head and torso elevated ~30° from horizontal), and after 3 min of standing [10, 11]. Tests during study drug treatment were performed approximately 3 h after drug administration; brachial arterial BP was recorded using a mercury, aneroid, or automated digital sphygmomanometer (the same type of device was used for each patient throughout the study).

### Safety/tolerability assessments

In each study, safety was assessed descriptively using reported adverse events (AEs), clinical laboratory values, vital signs, and electrocardiography.

### Statistical analyses

Efficacy analyses included data from randomized treated patients (the pooled full analysis set), with data from week 1 of randomized treatment. Mean values for the change from baseline (defined as the last non-missing value before the first dose of study treatment) to week 1 of randomized treatment in the droxidopa and placebo groups were compared using the Wilcoxon rank sum test. Missing data were imputed using the last observation carried forward, and statistical significance was set at the 2-sided 5% level. The same model was employed to assess changes in the OHQ composite score from randomization to the end of the study in subsets of patients defined by primary diagnosis, age (<65, ≥65, and ≥75 years), sex, and use of dopa decarboxylase inhibitors (DDCIs) during the study. For BP data, the mean change from baseline (before any exposure to droxidopa) to the end of the study was assessed using the Wilcoxon rank sum test, but with missing data excluded. For CGI-S scores, the comparisons of the relative distribution of patients treated with droxidopa or placebo were performed using the Fisher exact test.

### Ethics and good clinical practice

Each study was conducted in full accordance with the Declaration of Helsinki and its amendments, the International Conference on Harmonisation Good Clinical Practice guidelines, and the laws and regulations of the countries in which the research was performed. Studies were approved by centralized or local institutional review boards, and patients provided written informed consent before study procedures began. The studies were registered with ClinicalTrials.gov (NCT00782340, NCT00633880, and NCT01176240) [10–13].

## Results

### Patient disposition

**Fig. 2:**
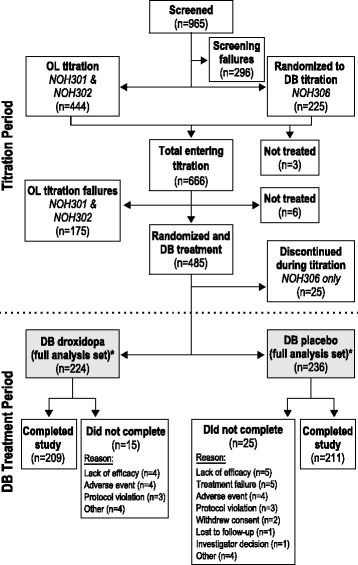
Patient disposition. DB = double-blind; OL = open-label. *Population used for the integrated analyses

Among all of the patients who entered the droxidopa dose optimization period (*n* = 666), 460 patients were evaluated in the double-blind study treatment period and were included in the full analysis set for these integrated analyses (Fig. 2). A total of 15 of 224 patients (6.7%) in the droxidopa group and 25 of 236 patients (10.6%) in the placebo group did not complete the double-blind portion of the trial. Reasons for discontinuation in the droxidopa group were lack of efficacy (4/15; 26.7%), AEs (4/15; 26.7%), protocol violations (3/15; 20.0%), and other (4/15; 26.7%). Reasons for discontinuation in the placebo group were lack of efficacy (5/25; 20.0%), treatment failure (5/25; 20.0%), AEs (4/25; 16.0%), protocol violations (3/25; 12.0%), withdrawn consent (2/25; 8.0%), lost to follow-up (1/25; 4.0%), investigator decision (1/25; 4.0%), and other (4/25; 16.0%). One patient was randomized to treatment with droxidopa but was erroneously administered placebo; consistent with intention to treat protocol, this patient is included in the droxidopa group for efficacy analyses and in the placebo group for baseline characteristics and safety assessment.

### Baseline characteristics

The demographic attributes, primary neurologic diagnoses, and baseline nOH parameters of the pooled treatment groups are summarized in Table 1. There were no marked differences in baseline characteristics or diagnoses between patients in the pooled droxidopa and placebo groups; patients had a significant symptom burden at baseline, as shown by scores on Item 1 of the OHSA (means, 6.0 and 5.8, respectively; Table 1).

**Table 1:** Baseline Characteristics and Baseline OHQ Scores^a^

Variable	Pooled Studies	
	Droxidopa	Placebo
Baseline characteristics		
N	225	235
Male sex, n (%)	132 (58.7)	142 (60.4)
White race, n (%)	220 (97.8)	221 (94.0)
Mean ± SD age, y	65.0 ± 14.7	65.4 ± 14.6
Primary clinical diagnosis, n (%)		
Parkinson disease	150 (66.7)	157 (66.8)
Pure autonomic failure	34 (15.1)	38 (16.2)
Multiple system atrophy	31 (13.8)	25 (10.6)
Nondiabetic autonomic neuropathy	4 (1.8)	9 (3.8)
Dopamine ß-hydroxylase deficiency	0	1 (0.4)
Other	6 (2.7)	5 (2.1)
Baseline OHQ scores		
Mean ± SD OHQ composite score (n)	5.9 ± 1.7 (224)	5.8 ± 1.9 (232)
Mean ± SD OHSA scores, n	225	235
Item 1	6.0 ± 2.2	5.8 ± 2.4
Item 2	4.3 ± 2.9	3.7 ± 2.9
Item 3	5.8 ± 2.5	5.6 ± 2.7
Item 4	5.9 ± 2.4	5.9 ± 2.6
Item 5	4.6 ± 2.7	4.7 ± 2.8
Item 6	4.0 ± 3.2	4.4 ± 3.3
Composite score	5.6 ± 1.7	5.5 ± 1.8
Mean ± SD OHDAS scores (n)		
Item 1	5.1 ± 2.8 (223)	5.3 ± 2.8 (230)
Item 2	7.0 ± 2.4 (214)	6.9 ± 2.8 (229)
Item 3	4.9 ± 2.9 (223)	4.7 ± 2.9 (228)
Item 4	6.7 ± 2.8 (211)	6.6 ± 3.0 (223)
Composite score	6.2 ± 2.0 (224)	6.1 ± 2.3 (231)

*OHDAS* Orthostatic Hypotension Daily Activity Scale, *OHQ* Orthostatic Hypotension Questionnaire, *OHSA* Orthostatic Hypotension Symptom Assessment

^a^One patient was randomized to receive droxidopa but was erroneously administered placebo in Study NOH301; this patient was placed among placebo recipients for baseline characteristics and for safety assessments, and among droxidopa recipients for efficacy analyses.

### Study drug dosage

**Fig. 3:**
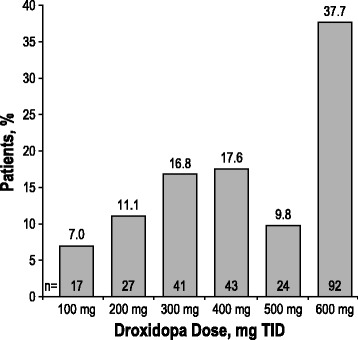
Frequency distribution of the optimized droxidopa dose in patients randomized to double-blind droxidopa (*n* = 244). TID = 3 times daily

The mean droxidopa dose during the double-blind administration period was 429 ± 163 mg, with 38% of patients (92/244) taking the maximum dosage of 600 mg 3 times daily (TID). The frequency distribution of the dose of droxidopa found to be optimal in patients randomized to treatment with droxidopa is shown in Fig. 3. By returned capsule counts, mean dosage compliance was >94% for droxidopa and placebo in all studies.

### Orthostatic hypotension questionnaire outcomes

**Fig. 4:**
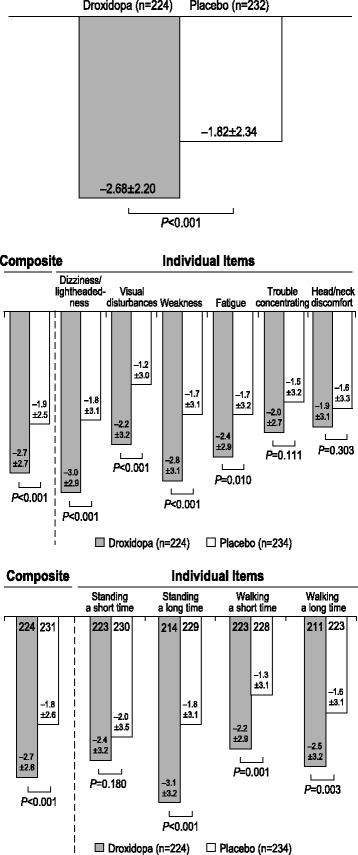
Mean score change* from baseline to week 1/EOS: (**a**) OHQ, (**b**) OHSA, and (**c**) OHDAS. EOS = end of study; OHDAS = Orthostatic Hypotension Daily Activity Scale; OHQ = Orthostatic Hypotension Questionnaire; OHSA = Orthostatic Hypotension Symptom Assessment. *Score change on a rating scale from 0 (none/no interference) to 10 (worst possible/complete interference). A negative change represents a decrease in symptom burden

Mean changes in OHQ items and composite scores from baseline to the end of the study are shown in Fig. 4a–c. Treatment with droxidopa was associated with a unit change of –2.68 ± 2.20 in the OHQ composite score; the difference was statistically significant compared with the change for placebo (−1.82 ± 2.34; *P* < 0.001; Fig. 4a). Treatment with droxidopa resulted in significant improvements in the OHSA, the OHQ domain that assesses nOH symptoms, including the composite score (*P* < 0.001), as well as 4 of the 6 individual items, including dizziness/lightheadedness (*P* < 0.001), visual disturbances (*P* < 0.001), weakness (*P* < 0.001), and fatigue (*P* = 0.010; Fig. 4b); the improvement in the remaining 2 symptom items did not reach statistical significance. Treatment with droxidopa also resulted in statistically significant reductions in the OHDAS, the OHQ domain that assesses activities of daily living, including the composite score (*P* < 0.001) and 3 of the 4 individual assessment categories (standing a long time [*P* < 0.001], walking a short time [*P* = 0.001], and walking a long time [*P* = 0.003; Fig. 4c]); the improvement in Item 1, standing a short time, did not reach statistical significance.

### Hemodynamic outcomes

**Fig. 5:**
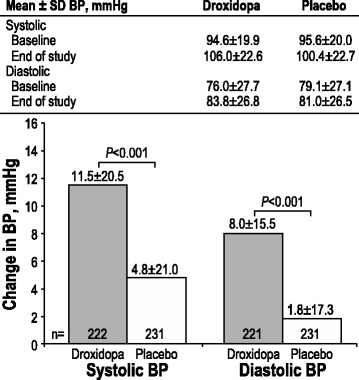
Changes in standing systolic and diastolic BP from baseline to week 1/EOS. EOS = end of study; BP = blood pressure

Treatment with droxidopa produced a significant increase in upright SBP compared with placebo (11.5 ± 20.5 vs 4.8 ± 21.0 mmHg; *P* < 0.001). A significant increase in upright DBP was also noted for droxidopa versus placebo (8.0 ± 15.5 vs 1.8 ± 17.3 mmHg; *P* < 0.001; Fig. 5).

### Clinical global impression outcome

**Fig. 6:**
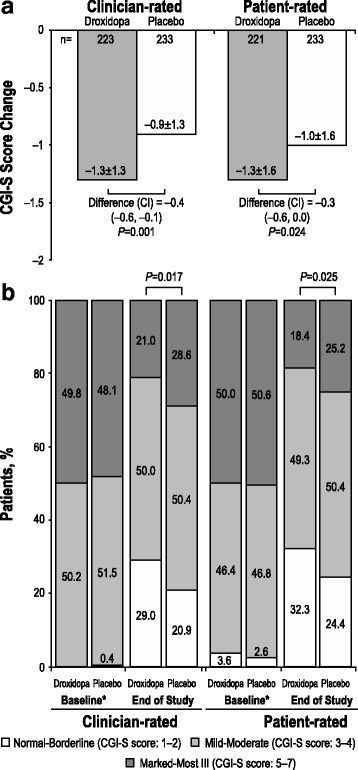
Changes in CGI-S scores* (**a**) and nOH severity categorization (**b**) from baseline to week 1/EOS. CGI-S = Clinical Global Impression–Severity; EOS = end of study; nOH = neurogenic orthostatic hypotension. *Score change on a rating scale from 1 (no symptoms) to 7 (severe symptoms)

The mean ± SD change from baseline to week 1 of randomized treatment in the CGI-S scores recorded by physicians was −1.3 ± 1.3 in the droxidopa group and −0.9 ± 1.3 in the placebo group. The adjusted mean difference from placebo was −0.4 (CI, −0.6 to −0.1; *P* = 0.001; Fig. 6a). Similar results were observed for the patient-rated CGI-S scores, with mean ± SD changes from baseline to week 1 of −1.3 ± 1.6 for patients in the droxidopa treatment group and −1.0 ± 1.6 for patients in the placebo group. The adjusted mean difference from placebo was −0.3 (CI, −0.6 to 0.0; *P* = 0.024).

At week 1 of randomized treatment, significant differences were also noted in the relative distributions of both the clinician-rated and patient-rated CGI-S score–based categorizations of nOH severity for droxidopa versus placebo (*P* = 0.017 and *P* = 0.025, respectively; Fig. 6b).

### Subgroup analyses

**Fig. 7:**
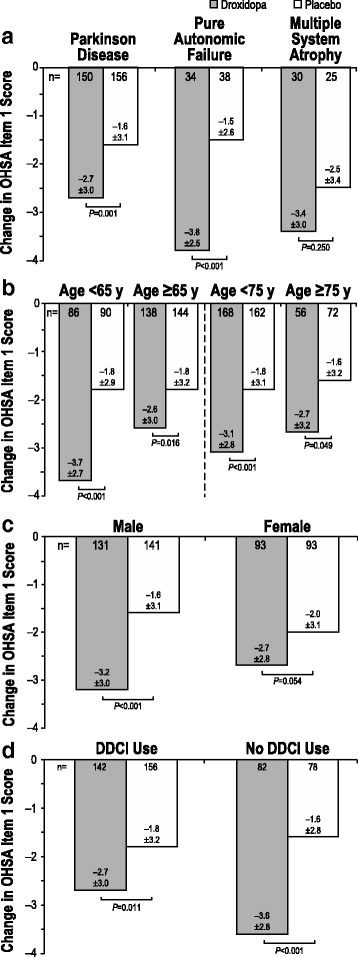
OHSA Item 1* score change: diagnosis (**a**), age (**b**), sex (**c**), and DDCI use (**d**). DDCI = dopa decarboxylase inhibitor; OHSA = Orthostatic Hypotension Symptom Assessment. *Score change on a rating scale from 0 (none) to 10 (worst possible). A negative change represents a decrease in symptom burden of dizziness/lightheadedness

Dizziness/lightheadedness while standing (measured by OHSA Item 1) is considered to be the cardinal symptom of nOH, and has been chosen by the FDA as the primary endpoint to assess the efficacy of nOH treatments. Therefore, we used this symptom to assess the effect of droxidopa in subpopulations of interest. Mean changes in OHSA Item 1 from baseline to the end of the study in patient subgroups based on diagnosis, age, sex, and DDCI use are shown in Fig. 7a–d. Droxidopa was significantly more effective than placebo in decreasing dizziness/lightheadedness symptoms in patients with a diagnosis of PD or pure autonomic failure (*P* ≤ 0.001; Fig. 7a). Among patients with MSA, treatment with droxidopa resulted in a larger decrease in symptom burden compared with patients with PD; however, this improvement was not statistically significant, apparently due to a greater placebo effect in this patient group. The numbers of patients in the other diagnostic groups were too small to draw conclusions. Further studies are required to determine whether patient characteristics at baseline (eg, plasma catecholamine levels, underlying diagnosis) can be used to predict greater treatment efficacy.

Treatment with droxidopa resulted in statistically significant improvements in OHSA Item 1 in all age stratification categories (aged <65 years vs ≥65 years; aged <75 years vs ≥75 years), although it appeared that the magnitude of the overall decrease in the score was lower among older patients compared with their younger counterparts (Fig. 7b). There also appeared to be an effect of sex on the efficacy of droxidopa, with improvement in OHSA Item 1 scores in women not reaching significance (−2.7 ± 2.8 vs −2.0 ± 3.1 for placebo, respectively; *P* = 0.054; Fig. 7c), whereas the same analysis for men was significant (−3.2 ± 3.0 vs −1.6 ± 3.1; *P* < 0.001).

Treatment with droxidopa resulted in statistically significant improvements in dizziness/lightheadedness compared with placebo, even in patients receiving concomitant DDCIs (*P* ≤ 0.011; Fig. 7d). However, the magnitude of the overall decrease in the scores was lower in patients receiving DDCIs compared with patients not receiving DDCIs (−2.7 ± 3.0 vs −3.6 ± 2.8, respectively). Therefore, to confirm the efficacy of droxidopa in patients receiving DDCIs, 2 complementary post hoc analyses were performed. A post hoc analysis of covariance, followed by post hoc pair-wise comparisons, did not show a significant difference between the symptomatic improvement in patients on droxidopa who were concomitantly taking DDCIs and those who were not (data not shown). Additionally, baseline characteristics were used to model a propensity score for each patient. This score (together with DDCI use, droxidopa/placebo treatment, and the DDCI-droxidopa treatment interaction) was used in a general additive model to confirm that there was no significant difference between droxidopa efficacy in patients taking DDCIs and patients not taking DDCIs, even after taking into account the presence of the DDCI and the DDCI-droxidopa treatment interaction. Thus, these results suggest that droxidopa can be titrated to doses that provide a beneficial effect even in patients taking clinically relevant doses of DDCIs. Of the patients randomized to receive treatment with droxidopa, those receiving DDCIs were given 439 ± 163 mg droxidopa TID, and those not receiving DDCIs were given 421 ± 162 mg TID.

### Safety

Adverse events reported during the double-blind treatment period are summarized in Table 2, separating studies with short-term exposure to droxidopa (Studies NOH301 and NOH302; 1–2 weeks) from the study evaluating longer exposure (Study NOH306, 8–10 weeks).

**Table 2:** Adverse Events Documented During Double-Blind Treatment

Variable	Pooled Studies NOH301 and NOH302		Study NOH306	
	Droxidopa	Placebo	Droxidopa	Placebo
N	131	132	114	108
Duration, wk	1–2		8–10	
Any AE, n (%)	30 (22.9)	31 (23.5)	91 (79.8)	87 (80.6)
Any severe AE, n (%)	0	2 (1.5)	10 (8.8)	9 (8.3)
Any AE leading to study drug discontinuation, n (%)	0	2 (1.5)	12 (10.5)	5 (4.6)
AE type,^a^ n (%)				
Headache	8 (6.1)	4 (3.0)	15 (13.2)	8 (7.4)
Dizziness	5 (3.8)	2 (1.5)	11 (9.6)	5 (4.6)
Urinary tract infection	4 (3.1)	2 (1.5)	4 (3.5)	5 (4.6)
Decreased appetite	2 (1.5)	1 (0.8)	1 (0.9)	0
Fatigue	2 (1.5)	3 (2.3)	8 (7.0)	6 (5.6)
Feeling abnormal	2 (1.5)	0	2 (1.8)	1 (0.9)
Hypertension	2 (1.5)	0	8 (7.0)	1 (0.9)
Nausea	2 (1.5)	2 (1.5)	10 (8.8)	5 (4.6)
Pyrexia	2 (1.5)	0		
Rhinorrhea	2 (1.5)	0		
Syncope	2 (1.5)	1 (0.8)	1 (0.9)	4 (3.7)
Falls	1 (0.8)	9 (6.8)	NA^b^	NA^b^
Edema peripheral	0	2 (1.5)	5 (4.4)	6 (5.6)
Loss of consciousness	0	3 (2.3)		
Vision blurred	0	2 (1.5)	1 (0.9)	1 (0.9)

*AE* adverse event, *NA* not applicable

^a^Classified using *Medical Dictionary for Regulatory Activities* preferred term.

^b^In Study NOH306, falls were recorded as an efficacy assessment.

In Studies NOH301 and NOH302, there were no obvious differences in AEs between groups, with the exception of an increase in headaches in the droxidopa group (6.1% vs 3.0% for placebo). Data from Study NOH306 with longer drug exposure also indicated that most AEs occurred at similar rates in the droxidopa and placebo groups. Compared with placebo, a greater proportion of patients receiving droxidopa reported AEs of headache (13.2% vs 7.4%), dizziness (9.6% vs 4.6%), nausea (8.8% vs 4.6), and hypertension (7.0% vs 0.9%).

The incidence of supine SBP >160, >180, and >200 mmHg (at all 3 measurements during the 10-min supine period of an orthostatic standing test) is shown in Table 3. There was a small increase in BP readings >160 mmHg in both groups.

**Table 3:** Incidence of Supine SBP >160, >180, and >200 mmHg by Double-Blind Treatment

	Pooled Studies NOH301 and NOH302				Study NOH306			
Variable	Droxidopa (*n* = 131)		Placebo (*n* = 132)		Droxidopa (*n* = 114)		Placebo (*n* = 108)	
	Baseline^a^	End of Study	Baseline^b^	End of Study	Baseline	Overall	Baseline	Overall
Duration of exposure, wk	1–2				8–10			
Observed supine SBP,^c^ n (%)								
> 160 mmHg	5 (3.8)	13 (9.9)	3 (2.3)	8 (6.1)	5 (4.4)	33 (28.9)	9 (8.3)	26 (24.1)
> 180 mmHg	0	4 (3.1)	0	2 (1.5)	0	9 (7.9)	2 (1.9)	5 (4.6)
> 200 mmHg	0	0	0	0	0	4 (3.5)	0	1 (0.9)

*SBP* systolic blood pressure

^a^ *n* = 130

^b^ *n* = 131

^c^At all 3 measurements during the 10-min supine period of an orthostatic standing test

In Studies NOH301 and NOH302, a lower incidence of falls reported as AEs was observed in the droxidopa group (0.8% vs 6.8% for placebo). In Study NOH306, the number of falls was prospectively collected as a secondary efficacy outcome. The data showed that the aggregate rate of falls per patient-week was 0.4 in the droxidopa group compared with 1.7 in the placebo group. Overall, 68% fewer falls were reported in patients treated with droxidopa compared with patients receiving placebo (total number of falls, 308 [droxidopa] vs 908 [placebo]).

## Discussion

Here, we report the analysis of the combined dataset of the 3 pivotal studies that support the efficacy and safety of droxidopa for the treatment of nOH in patients with chronic autonomic failure. Combined, these studies represent the largest randomized clinical trial experience in the treatment of nOH (a total of 460 patients) and provide a unique opportunity to learn about the condition and its treatment with droxidopa.

Consistent with findings of the individual studies, the combined analyses showed that droxidopa significantly reduced the cardinal symptom of nOH, orthostatic dizziness/lightheadedness, feeling faint or feeling like you might black out (the primary outcome recommended by the FDA), by −3.0 ± 2.9 units versus 1.8 ± 3.1 units for placebo (*P* < 0.001). Furthermore, the combined analysis provided increased power to detect the effect of droxidopa on the less frequently reported symptoms of nOH and on symptom impact on activities of daily living. Droxidopa significantly improved 3 of the other 5 individual measures of orthostatic symptoms (visual disturbances [*P* < 0.001], weakness [*P* < 0.001], and fatigue [*P* = 0.010]). Conversely, OHSA Items 5 (trouble concentrating) and 6 (head/neck discomfort) were not particularly useful in discriminating between the treatment groups. Droxidopa also significantly improved 3 of the 4 individual measures of nOH symptoms: interference with the ability to carry out activities of daily living (standing a long time [*P* < 0.001], walking a short time [*P* = 0.001], and walking a long time [*P* = 0.003]); the improvement in OHDAS Item 1, standing a short time, did not reach statistical significance. Overall, the pooled data show that patients treated with droxidopa derived a broad range of symptomatic benefits. Importantly, it appears that these symptom improvements translated into an increased ability of patients to perform activities of daily living.

Clinical improvements in symptoms of nOH and in symptom impacts were seen in all 3 studies, and in 2 of the 3 studies, the improvements were statistically significant. Nonetheless, proving treatment efficacy was more challenging than we had expected; recognition of these challenges will inform the design of future randomized clinical trials in patients with nOH. Several factors may explain this difficulty in addition to the relatively small size of the studies, a consequence of the challenges in recruiting adequate numbers of patients with this orphan condition. First, there was a large placebo effect. Because many of the outcome variables were subjective symptom scores, this finding is perhaps not surprising. However, even upright BP improved in patients randomized to placebo, raising the possibility that participation in the studies improved patients’ adherence to general non-pharmacologic treatment recommendations for nOH (increased salt and water intake, compression garments, and sleeping with the head of the bed elevated).

Second, there was significant variability in the quantification of symptoms of OH. This is likely due a combination of factors, including the subjective nature of an unanchored scale such as the OHQ, bias in the patient’s recall of symptoms occurring in the past week toward significant individual events (such as a bad or good day), and shifts in the perception/recollection of baseline condition as a response to improvement (i.e., the “treadmill effect” [16]), which can result in underestimation of the overall magnitude of the reported benefit.

Third, there was significant variability in the magnitude of individual patients’ response to droxidopa. Because patients with nOH have varying degrees of blunted baroreflex buffering, depending on the underlying diagnosis and severity of disease, these findings are not unexpected.

In these clinical trials, treatment with droxidopa was initiated at 100 mg TID and was escalated in 100-mg TID increments every 24 to 48 h, while monitoring BP for safety. The integrated data suggest that simply asking patients to grade their symptom burden, using OHSA Item 1 (dizziness, lightheadedness, feeling faint or feeling like you might black out), can be used to assess efficacy. As with other treatments, droxidopa is not universally effective; 8.7% of enrolled patients were unable to complete the dose optimization phase because of AEs or hypertension. Almost 40% of patients reached the maximal approved dose (600 mg TID). It is possible that some patient would have benefited from larger doses; however, the safety and efficacy of higher doses have not been tested.

With regard to post hoc subset analyses, treatment with droxidopa appears to be effective in all primary diagnosis subsets (PD, MSA, and pure autonomic failure). It should be noted that the results for patients with MSA were not significantly different from placebo; however, this could be due to a larger than expected placebo effect and the small sample size for the MSA group (*n* = 55). These reasons may also explain the differences noted by sex (i.e., droxidopa appearing to be less effective in women, although a strong trend was still noted [*P* = 0.054]).

The efficacy of droxidopa in the subset of patients taking a DDCI is of particular interest because dopa decarboxylase (l-aromatic amino acid decarboxylase), the enzyme that converts droxidopa into active norepinephrine, also converts levodopa to dopamine. Inhibitors of this enzyme are routinely used together with levodopa to reduce the side effects associated with peripheral conversion of levodopa to dopamine in the treatment of PD. Thus, the pharmacologic effects of DDCIs should render droxidopa ineffective. Indeed, a previous proof-of-concept study found that a single 200-mg dose of the DDCI carbidopa prevented the pressor effects of a dose of droxidopa administered 90 min later [17]; however, this dose is 8- to 10-fold higher than those used in combination with levodopa for the treatment of PD. Another study found that DDCIs, at doses used clinically, did not prevent the therapeutic effect of droxidopa [18]. Similarly, it is reassuring that droxidopa produced significant symptomatic improvement in patients receiving a DDCI in our studies. The effect, however, seems to be lower than in patients not receiving a DDCI. Information on the dosage of DDCIs was not collected in the studies but it is likely that the effect is dose dependent. It is possible that patients receiving high doses of DDCIs may have a reduction in the pressor effect of droxidopa and may require titration to higher doses [9]. Nonetheless, in these studies, the average dose of droxidopa in patients receiving DDCIs (439 mg TID) was not markedly higher than in those not receiving DDCIs (421 mg TID). Nonetheless, when modifying dosages of levodopa/carbidopa in patients with PD, it may be necessary to re-titrate droxidopa.

There is less information about other drug-drug interactions that can potentiate the effects of droxidopa, but it is reassuring that drugs commonly used in the treatment of patients with PD were allowed in these trials. Nevertheless, any medication that reduces the metabolism of norepinephrine could theoretically potentiate the pressor effects of droxidopa [9]. There is limited experience with NRIs in these studies; only 28 patients received concomitant treatment with an NRI. Although there was no increase in the number of cardiovascular AEs reported in patients receiving concomitant NRIs, there remains the possibility that these drugs will potentiate the actions of droxidopa, which is converted to norepinephrine. However, this may be less critical when titrating droxidopa in patients on stable doses of NRIs or other drugs that reduce the metabolism of norepinephrine. Even so, care should be taken when adding NRIs to the treatment regimen of patients already receiving droxidopa.

Our results also underscore the need to develop objective endpoints to assess symptomatic burden in nOH. In the past, an increase in upright BP has been used as a surrogate for reduced symptom burden. A recent study in patients with PD noted that the presence of symptomatic OH was associated with an upright mean BP less than 75 mmHg [19]. In our studies, treatment with droxidopa resulted in improved upright BP; however, our patient population was less homogeneous. There was a significant relationship between symptoms and BP at a population level; however, the individual variation in both BP and OHQ scores made it impossible to predict symptomatic benefit for any given patient based on BP (data not shown).

Droxidopa appears to be well-tolerated. In the pooled analysis of studies with short-term exposure to droxidopa (Studies NOH301 and NOH302; 1–2 weeks), there were no obvious differences in AEs between groups, with the exception of headaches in the droxidopa group (6% vs 3% for placebo). In the study with a longer duration of drug exposure (Study NOH306; 8–10 weeks), headaches, dizziness, nausea, and hypertension were more frequently reported by patients receiving droxidopa, and the incidence of supine hypertension was greater with droxidopa. Although this finding is not unexpected, caution is required for patients with severe supine hypertension (who were excluded from these studies). Patients should be advised against lying down after taking droxidopa, and, instead of using a rigid TID regimen, the last dose should be given at least 3 h before bedtime. During the individual studies, no evidence of significant cardiac AEs and no trend for clinically meaningful electrocardiogram changes were noted; heart rate increases were minimal (approximately 1 beat per minute). Interestingly, the typical pattern of AEs seen with a selective alpha-1 agonist (i.e., piloerection, dysuria, and pruritus) [20] were not noted with treatment with droxidopa, suggesting that the mechanism of droxidopa involves more than just alpha agonistic effects.

It should be noted that these studies only enrolled patients with nOH associated with alpha synucleinopathies (pure autonomic failure, PD, and MSA), NDAN, and DBHD. The efficacy of droxidopa has not been studied in the treatment of OH due to other causes. Also, evidence of efficacy was limited to 1–2 and 8–10 weeks in these studies, although longer-term safety and efficacy data have been reported [21, 22]. Droxidopa received accelerated FDA approval, with commitment from the sponsor to undertake a postmarketing trial to assess the durability of efficacy; such a study is underway [23]. Future research can also determine if droxidopa is efficacious on other conditions characterized by central or peripheral deficits of norepinephrine.

## Conclusions

In summary, the results of this pooled analysis support the clinical benefit of droxidopa in the treatment of nOH and suggest that benefits extend beyond the improvement in the cardinal symptom of nOH, dizziness/lightheadedness, to include improvements in activities of daily living. This novel treatment, based on replenishment of norepinephrine, was effective in reducing symptom burden and improving upright BP. A wide variability of optimal doses was found to be effective, highlighting the need to individualize treatment in this patient population by careful titration of the dose, using symptomatic improvement as efficacy endpoints, and BP as safety endpoints. Overall, treatment with droxidopa was well-tolerated; however, caution should be used when treating patients with supine hypertension. Information gathered from this analysis will help guide future studies of nOH.

## Abbreviations

AE: Adverse event
BP: Blood pressure
CGI-S: Clinical Global Impression–Severity
DBHD: Dopamine β-hydroxylase deficiency
DBP: Diastolic blood pressure
DDCI: Dopa decarboxylase inhibitor
FDA: US Food and Drug Administration
MSA: Multiple system atrophy
NDAN: Nondiabetic autonomic neuropathy
nOH: Neurogenic orthostatic hypotension
NRI: Norepinephrine reuptake inhibitors
OH: Orthostatic hypotension
OHDAS: Orthostatic Hypotension Daily Activity Scale
OHQ: Orthostatic Hypotension Questionnaire
OHSA: Orthostatic Hypotension Symptom Assessment
PD: Parkinson disease
SBP: Systolic blood pressure
TID: 3 times daily
